# Supplementary material for: Oxidative Stress as a Contributor to Insulin Resistance in the Skeletal Muscles of Mice with Polycystic Ovary Syndrome
Source: Int J Mol Sci. 2022 Sep 27;23(19):11384. doi: 10.3390/ijms231911384 (PMC9569700; doi:10.3390/ijms231911384)
Supplement: Supplementary file 1 [file ijms-23-11384-s001.zip › ijms-1911191-supplementary.pdf]

Supplementary Materials:

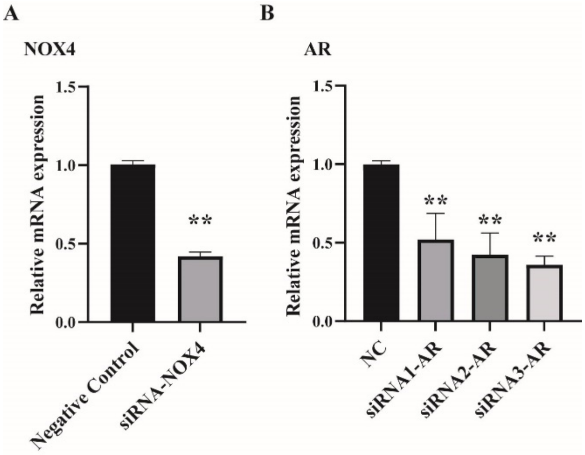

Supplemental Figure S1. Determination of the efficiency of siRNA-NOX4 and siRNA-AR in C2C12 cells. Fully differentiated C2C12 cells were transfected with siRNA-NOX4, siRNA-AR, or non-silencing RNA (NC). Thirty-six hours later, cells were collected and transfection efficiency was determined. (**A**) quantitative PCR assays of NOX4 mRNA levels. (**B**) quantitative PCR assays of *AR* mRNA levels. Three siRNA-AR sequences were used, designated siRNA1-AR, siRNA2-AR, and siRNA3-AR. The data are presented as means ± SEM of three independent experiments. *, $p < 0.05$, **, $p < 0.01$, T, testosterone. AR, androgen receptor.

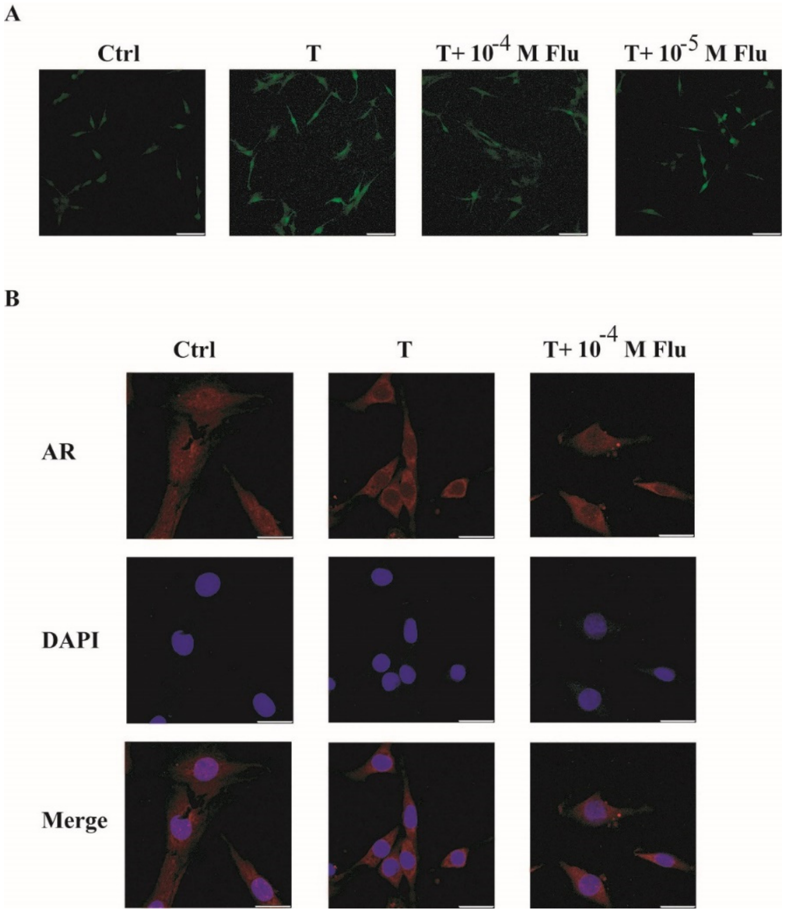

Supplemental Figure S2. Effects of flutamide intervention on T-treated C2C12 cells. (**A**) Representative photomicrographs of intracellular ROS in C2C12 cells.

Fully differentiated C2C12 cells were pretreated with flutamide (10^{-4} and 10^{-5} M) for 1 h followed by the treatment with T (0, 10^{-5}M) for 12 h. ROS was measured by DCFH-DA staining. Sale bar = 100 μm. (**B**) The subcellular localization of AR. Fully differentiated C2C12 cells were pretreated with flutamide (10^{-4} M) for 1 h followed by the treatment with T (0, 10^{-5}M) for 12 h. Red: AR; Blue: DAPI. Scale bar = 25 μm. T, testosterone; AR, androgen receptor; Flu, flutamide.

Supplemental Table S1. Primer sequences used for quantitative RT-PCR

Gene	Sequences 5′-3′
GAPDH	F: ATGACATCAAGAAGGTGGTG<br>R: CATACCAGGAAATGAGCTTG
AR	F: AAGGGAGGTTACGCCAAAGG<br>R: TGCCGTAGTCCAATGGGTTC
NOX4	F: TGTTGGGCCTAGGATTGTGTT<br>R: AGGGACCTTCTGTGATCCTCG
SOD1	F: CCAGTGCAGGACCTCATTTT<br>R: TTGTTTCTCATGGACCACCA
SOD2	F: TCTGCAGGGTACAACCATCA<br>R: ACCTCCATCGGGTTGTAGTG
GR	F: TCCAAGTGGTGACTTCCGTG<br>R: ATGTGGCCCTTTTCATCCGT
NOX2	F: TGTGGTTGGGGCTGAATGTC<br>R: CTGAGAAAGGAGAGCAGATTTCG
Catalase	F: CACTGACGAGATGGCACACT<br>R: TGTGGAGAATCGAACGGCAA
GPx1	F: ACAGTCCACCGTGTATGCC<br>R: TCATTCTTGCCATTCTCCTGGTG

Supplemental Table S2. siRNA sequences targeting AR and NOX4 mRNA

	siRNA Sequences 5′-3′
siRNA1-AR	F: GACUCAGCUGCCCCAUCCATT
	R: UGGAUGGGGCAGCUGAGUCTT
siRNA2-AR	F: CUGGGAAAGUCAAGCCCAUTT
	R: AUGGGCUUGACUUUCCCAGTT
siRNA3-AR	F: CCUUUGCUGCCUUGUUAUCUATT
	R: UAGAUAACAAGGCAGCAAAGGTT
siRNA-NOX4	F: GACCUGACUUUGUGAACAUTT
	R: AUGUUCACAAAGUCAGGUCTT
Negative control (NC)	F: UUCUCCGAACGUGUCACGUTT
	R: ACGUGACACGUUCGGAGAATT
